# Addressing methodological challenges in multiple long-term conditions research: A stakeholder workshop using a nominal group technique method

**DOI:** 10.1177/26335565251372222

**Published:** 2025-09-24

**Authors:** Hajira Dambha-Miller, Glenn Simpson, Lucy Smith, James Finney, Salwa S. Zghebi, Sarah E. Hughes, Victoria L. Keevil, Ge Yu, Clare MacRae, Kamlesh Khunti, Colin McCowan

**Affiliations:** 1 Primary Care Research Centre, 12211 University of Southampton, Southampton, UK; 2 Leicester Diabetes Centre, 4488 University of Leicester, Leicester General Hospital, Leicester, UK; 3 Centre for Primary Care and Health Services Research, 5292 The University of Manchester, Manchester, UK; 4 Centre for Patient Reported Outcome Research, 1724 University of Birmingham, Birmingham, UK; 5 Department of Medicine, School of Clinical Medicine, 2152 University of Cambridge, Cambridge, UK; 6 Institute of Psychiatry, Psychology & Neuroscience, 4616 King’s College London, London, UK; 7 Advanced Care Research Centre, 151027 University of Edinburgh, Edinburgh, UK; 8 School of Medicine, 7486 University of St Andrews, St Andrews, UK

**Keywords:** multiple long-term conditions, research methodologies, collaboration, workshop

## Abstract

**Background:**

Multiple long-term conditions (MLTC) - which refer to the coexistence in an individual of two or more long-term conditions - are a growing global concern, causing significant strain on healthcare systems and increasing care costs. Research into MLTC is a strategic priority for healthcare services, policymakers and research funders.

**Methods:**

To address these complexities, the UK’s National Institute for Health and Care Research (NIHR) established the MLTC Cross-NIHR Collaboration (MLTC CNC) programme, to foster interdisciplinary collaboration and address key gaps in MLTC research. As part of this initiative, the Methodologies Workstream organised a two-day stakeholder workshop in March 2024 aimed at identifying current methodological challenges in MLTC research, prioritising key areas for improvement, and developing strategies to enhance research methodologies. The workshop employed a participatory and iterative approach, using structured presentations, facilitated group work, and the Nominal Group Technique (NGT) to promote cross-disciplinary collaboration and achieve consensus on key research priorities for MLTC.

**Results:**

Twenty-three delegates attended the workshop from a range of institutions and sectors, including representatives from data science, epidemiology, clinical trials, quality improvement, social sciences, healthcare management, clinical practice, industry, patient advocacy groups, policymakers, patients, carers, and public representatives. The workshop identified critical knowledge gaps in MLTC research methodologies, including challenges with disease classification, data integration, analytical approaches, and the inclusion of diverse population subgroups.

**Conclusion:**

By addressing these methodological gaps and fostering collaboration across disciplines, the MLTC research community can generate more rigorous, inclusive, and impactful evidence, ultimately improving healthcare delivery and patient outcomes.

## Background

Multiple long-term conditions (MLTC) or multimorbidity, refers to the co-existence of two or more chronic health conditions in an individual and represents a growing global challenge for healthcare systems.^1^ The rising prevalence of MLTC has profound implications for patient outcomes, healthcare service utilisation, and social care costs.^2^ For instance, healthcare costs for individuals with MLTC are multiple times higher compared to those with a single chronic condition.^3^ Moreover, the co-occurrence of mental health conditions with physical illnesses further compounds the burden, often leading to poorer health outcomes and increased service usage.^4^ As such, addressing the challenges posed by MLTC has become a priority for national health research agendas, including that of the United Kingdom’s National Institute for Health and Care Research (NIHR).^5^ The MLTC Cross-NIHR Collaboration (MLTC CNC) programme was established to foster coordinated efforts among researchers and research organisations across biomedical and applied settings to address pressing questions surrounding MLTC. A component of the MLTC CNC programme is the Methodologies Workstream, which seeks to tackle methodological challenges in two key areas: (i) the prevention and treatment of MLTC and (ii) the management of MLTC and its consequences. The workstream aims to enhance measurement standards, promote research transparency and reproducibility, and build methodological capacity. By addressing these challenges, the programme seeks to support the development of scalable and effective interventions to improve the quality of life for individuals living with MLTC.

Current research approaches to MLTC face significant limitations due to the heterogeneous nature of disease combinations and the varying trajectories and outcomes across different demographic and social groups.^6^ In the UK, common disease combinations include cardiovascular diseases and diabetes or the concurrent presence of mental and physical health conditions.^7^ These challenges are compounded by inconsistent measurement frameworks, limited methodological transparency, and a lack of standardised approaches, all of which impede cross-study comparability and the development of generalisable solutions.^8^ There is therefore an urgent need to systematically identify research gaps, prioritise methodological challenges, and develop robust frameworks to support the standardisation of MLTC research methodologies. To advance these aims, the Methodologies Workstream convened a two-day workshop involving a diverse group of stakeholders with the aim of facilitating a collaborative exploration of key challenges in MLTC research methodologies and identifying actionable steps for improvement. This paper summarises the key outcomes of the workshop, offering insights into existing knowledge gaps, priorities for methodological development, and recommendations to address current limitations. The findings are intended to inform future research strategies, ultimately contributing to a more robust and standardised approach to MLTC research and improving outcomes for patients and care systems.

## Methods

### Workshop overview

In March 2024, a two-day in-person stakeholder workshop was convened in Leicester to engage experts from varied disciplines and backgrounds in identifying and addressing key gaps in current research methodologies for Multiple Long-Term Conditions (MLTC) in the UK (Appendix 1 & Appendix 2). Workshops of this nature are increasingly employed in healthcare research to foster cross-disciplinary collaboration and generate innovative solutions for complex, multifaceted issues.^9^ Delegates were invited through an open call disseminated across the UK research community via established NIHR networks (https://www.nihr.ac.uk/about-us/what-we-do/multiple-long-term-conditions/collaboration), academic institutions, social media channels, and targeted emails to key stakeholder organisations (Appendix 3). This multi-channel approach was designed to maximise the diversity of expertise and experience among participants, ensuring that different perspectives were represented. The invited delegates were selected based on their expertise, experience in MLTC research or involvement in healthcare services related to MLTC. Two individuals with lived experience of MLTC and/or caring responsibilities were recruited via the open call. They participated on equal footing alongside other delegates and were reimbursed in line with NIHR guidance.

### Workshop design and structure

The workshop was facilitated by four experts in participatory research methodologies, two of whom were also experts in MLTC research. Facilitators ensured discussions remained structured, inclusive, and goal-oriented, enabling productive exchanges across disciplines. Facilitators actively encouraged contributions by creating an open and inclusive environment whereby all attendees were invited to share their perspectives. They used targeted questions to guide the dialogue, ensuring that discussions remained focused on key issues while allowing for diverse input. Throughout the sessions, facilitators synthesised group outputs in real-time, summarising key points, identifying common themes, and ensuring that actionable recommendations were clearly articulated. This process involved regular check-ins with the groups to refine ideas and align them with the workshop’s goals, ensuring that the discussions resulted in concrete and actionable outcomes. It was designed to facilitate both broad contextualisation and focused exploration of methodological challenges. A participatory and iterative approach was adopted, incorporating structured presentations, facilitated group work, and collaborative problem-solving techniques. This design aligns with established scientific workshop methodologies that prioritise multi-stakeholder engagement and systematic exploration of complex research challenges.^10^ We followed an adaptation of the Nominal Group Technique (NGT), often used in research settings to structure decision-making processes and achieve consensus on key priorities.^11^ NGT involves a series of phases including problem identification, generation of ideas and potential solutions, group discussion and individual ranking, making it especially useful in addressing complex issues in health research.^12^ The format was divided into two main sessions to ensure a balance between contextual framing and focused problem-solving activities.

### Session 1: Contextualisation and thematic identification

The initial session featured presentations by subject experts who outlined the current challenges in MLTC research and the existing evidence in this field. Facilitators provided structured presentations ahead of discussions to set the context and frame the key challenges in MLTC research. These presentations were designed to ensure participants had a shared understanding of the current landscape and the gaps in research methodologies. The presentations covered both theoretical frameworks and practical considerations, laying the groundwork for focused discussions. By providing this foundational knowledge at the beginning, facilitators ensured that the subsequent discussions were grounded in a common understanding, enabling more targeted problem-solving and idea generation among participants. The objective of the session was to provide a shared understanding of participants’ experiences and perceptions regarding methodological barriers, creating a foundation for subsequent discussions.

Participants then engaged in a brainstorming exercise designed to elicit diverse perspectives on current methodological gaps. Following the brainstorming phase, a thematic clustering process was employed, where ideas were categorised into preliminary themes by facilitators and refined through participant feedback. This iterative refinement process ensured alignment and consensus among participants, consistent with participatory research best practices.^13^ By the end of Session 1, three primary themes for further exploration were identified. These themes were derived directly from discussions and reflected participants’ insights on major obstacles in conducting MLTC research. The iterative theme refinement approach adheres to best practices for workshops addressing complex challenges.^14^

### Session 2: Thematic exploration and solution development

In the second session, delegates were divided into three groups, each tasked with exploring one of the identified themes in greater depth. The rationale for dividing participants into three groups was to facilitate more focused and manageable discussions, allowing for in-depth exploration of specific aspects and to balance the groups within the available facilitators. Facilitators pre-assigned the groups based on the participants’ expertise and experience in different sectors to maximise the diversity of knowledge and viewpoints within each group, promoting a well-rounded discussion. Each group was facilitated using a structured discussion guide to ensure the systematic exploration of specific methodological challenges and the co-development of actionable solutions (Appendix 4).

The discussions followed a structured iterative approach, to encourage idea generation and refinement.^15^ Group facilitators ensured balanced participation by all members to contribute and guided the conversation to maintain focus. Each group employed structured techniques, such as issue mapping and root cause analysis, to dissect methodological challenges and generate robust, evidence-informed recommendations.^16,17^ Facilitators ensured productive and interactive group discussions, including that these were equitable and accessible to every participant. They encouraged and supported contributions from all attendees, guided the dialogue with targeted questions, and synthesised group outputs into actionable recommendations. This approach reflects established facilitation techniques based on active moderation and inclusive engagement.^18^

### Data collection and analysis

Detailed qualitative data were collected through multiple methods, including facilitator notes and real-time documentation of key points on shared visual aids such as flip charts and digital whiteboards. Following the workshop, the collected data were transcribed and subjected to thematic analysis. The analysis by qualitative researchers involved an initial open coding process to identify recurring ideas and patterns, followed by axial coding to group related themes and develop higher-order categories.^19^ Three qualitative researchers independently assessed the codes to ensure consistency and reliability in the analysis. The thematic clustering process in the workshop was designed to identify and organise key research priorities by grouping similar ideas and themes that emerged during the group discussions. After participants generated ideas and potential solutions, the facilitators used a systematic approach to categorise these contributions into coherent clusters. This was achieved through group discussions and individual ranking, where participants reviewed the identified themes, refined them, and categorised them into broader thematic areas. The Nominal Group Technique provided a structured framework for this process, ensuring that themes were discussed, prioritised, and agreed upon collectively.^20^ This approach ensured that insights from the workshop were comprehensively captured and rigorously analysed. To enhance the credibility and validity of the findings, member checking was employed, whereby workshop participants reviewed and validated the thematic analysis. This method ensured that the results accurately reflected the participants’ contributions and insights.^21^ Our systematic approach to data collection and analysis ensured a rigorous and transparent synthesis of the workshop outputs (Appendix 5).

## Results

Twenty-three delegates attended the workshop from a range of institutions and sectors, including representatives from data science, epidemiology, clinical trials, quality improvement, social sciences, healthcare management, clinical practice, industry, patient advocacy groups, policymakers, patients living with MLTC, carers, and public representatives. These diverse participants facilitated a comprehensive and interdisciplinary discussion on MLTC research methodologies. The three primary themes identified in session 1, along with the in-depth discussions that took place to refine and expand upon these themes in session 2, are described below.

1. Identifying Gaps in Methodological Understanding

Within this overarching theme, we identified three sub-themes. First, a primary focus of the discussions was the need for greater clarity and consensus regarding how MLTC should be defined and measured. Participants highlighted significant variability in the number and selection of conditions included in MLTC studies, leading to inconsistencies in prevalence estimates. For example, some MLTC studies include long COVID and chronic fatigue syndrome, whereas others focus primarily on core cardiometabolic conditions such as diabetes, hypertension, and heart disease.^22–27^ These variations in condition selection contribute to differing estimates of MLTC prevalence. Some studies defined MLTC as two or more chronic conditions, while others included different classifications depending on study objectives, data availability, and population characteristics.^28–30^ There was broad agreement that standardised condition lists, such as those developed through international Delphi consensus studies,^31^ should be more widely adopted to ensure comparability across research studies.

Secondly, another methodological challenge discussed was the use of different data sources in MLTC research, ranging from primary care records to survey data and hospital admission records as well as including populations with certain conditions such as those with diabetes or hypertension. Participants agreed that the variability in data sources contributed to discrepancies in research findings and that greater transparency is needed in reporting data selection criteria. It was suggested that guidelines should be developed to help researchers navigate these choices, particularly in studies focusing on MLTC prevalence, clustering of conditions, and associations with health outcomes.

The third sub-theme raised was the need for greater consideration of disease severity as a crucial factor in MLTC research. While some studies account for the severity of individual conditions, many do not, potentially leading to misleading conclusions about disease burden and its impact on health outcomes.^32–34^ For instance, a treatable skin cancer diagnosis does not carry the same implications as an end-stage metastatic cancer, yet both may be classified as ‘cancer’ under some MLTC definitions. Such discrepancies can obscure the real burden of disease and complicate the assessment of healthcare needs. There was consensus that future research should incorporate validated severity indices and consider how different condition combinations influence overall disease burden.

2. Promoting Transparency, Standardisation, and Reproducibility in MLTC Research

The recognition that MLTC research requires enhanced transparency in reporting methodologies and findings was highlighted. Participants supported the development of standardised reporting guidelines tailored to MLTC research, akin to the EQUATOR Network guidelines for health research reporting.^35^ Such guidelines would specify and help embed best practices for data collection, variable definitions, and analytical approaches, etc, to improve the reporting of these areas, thereby supporting efforts to enhance consistency and reproducibility in this field.

Further, the workshop highlighted the need for standardised outcome measures. This is particularly important to ensure comparability across studies and reduce the variability of research in this field. Ensuring alignment between these outcome measures and patient-prioritised metrics was recognised as a crucial step forward. Aligning these outcome measures with patient-prioritised metrics was also identified as an important step in advancing MLTC research.

Data sharing and collaboration were also identified as critical enablers of high-quality MLTC research. Participants recommended the establishment of a dedicated MLTC data repository, where researchers could share datasets, methodological frameworks, and analytical code to enhance collaboration and avoid duplication of efforts. However, several data-sharing barriers were noted, including privacy concerns, ethical considerations, and the logistical challenges of obtaining data-sharing agreements. To address these issues, the development of a UK national MLTC data repository or registry was proposed, with appropriate governance structures to ensure data security and ethical compliance,^36^ although we note that some similar repositories have already been established, such as the HDRUK and BHF Data science centre.^37,38^

3. Building Methodological Capacity in MLTC

The final discussions centred on strategies to build methodological capacity within the MLTC research community. Participants emphasised the importance of training and mentorship programmes to support early-career researchers (ECRs) and ensure the sustainability of expertise in MLTC research. In addition to traditional mentorship schemes, reverse mentorship models -where junior researchers introduce senior academics to emerging methodologies such as artificial intelligence (AI) and health technology applications - were suggested as innovative approaches to fostering interdisciplinary learning.

Collaboration between academic and non-academic stakeholders was also viewed as vital for ensuring that MLTC research remains relevant to real-world healthcare challenges. Industry partners, particularly small and medium-sized enterprises (SMEs), were identified as potential contributors to methodological innovation in MLTC research. Participants called for greater integration of policymakers and healthcare providers into research collaborations to ensure that MLTC methodologies align with practical healthcare needs.

The lack of a dedicated professional network for MLTC researchers was considered a major limitation in the field. Unlike disease-specific research communities, such as oncology or cardiovascular research, MLTC researchers often work in isolation or as part of broader multidisciplinary teams. To address this, participants proposed the creation of an online MLTC research network and a resource hub to facilitate knowledge exchange, funding updates, and methodological advancements. Establishing a platform for researchers to share best practices, funding opportunities, and methodological guidance was seen as a necessary step to advance MLTC research as a distinct field.

**Figure 1.**
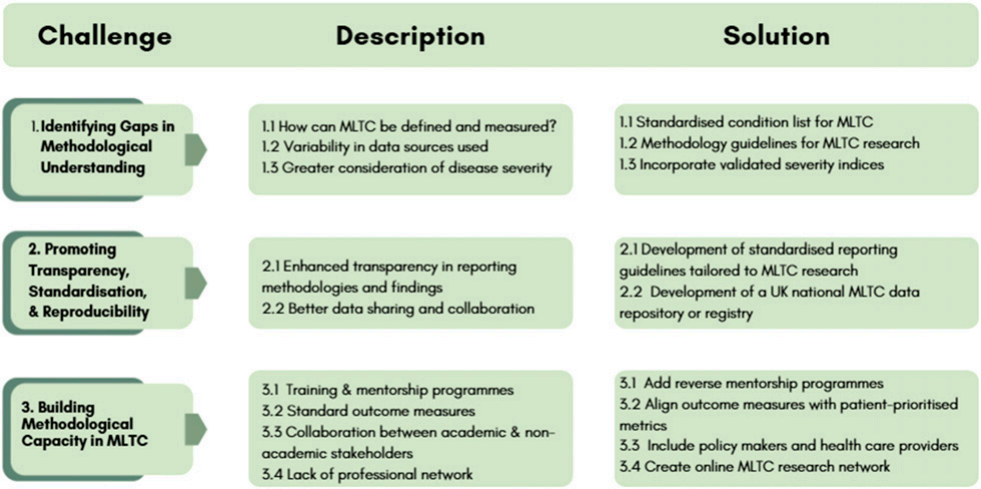
Summary of the key themes and solutions in MLTC research.

A summary of the key themes and solutions are shown diagrammatically below Figure 1.

## Discussion

This stakeholder workshop provided valuable insights into the methodological challenges in MLTC research. By bringing together experts from multiple disciplines, healthcare providers, patients and their family carers, and public representatives, the workshop identified key priority areas for methodological improvements. The findings add to the ongoing discussions in the literature on MLTC research, highlighting the need for standardisation, transparency, and methodological capacity building. A major theme to emerge from the discussions was the need for clearer definitions and standardised measurement approaches in MLTC research. The lack of consensus on MLTC definitions has long been recognised as a challenge.^7^ Workshop participants reiterated that inconsistencies in condition selection contribute to variability in prevalence estimates, as seen in studies that define MLTC using different disease clusters, such as long-COVID and chronic fatigue syndrome versus core cardiometabolic conditions. Similar concerns have been raised by the James Lind Alliance,^39^ which highlight the importance of structured, operational definitions for advancing MLTC research.

The need for standardised outcome measures was also highlighted, a topic of increasing focus in the field.^40^ Recent efforts to define core outcome sets for multimorbidity research aim to improve comparability across studies, but they also introduce variability depending on the research context.^41^ Ensuring alignment between these outcome measures and patient-prioritised metrics was recognised as a crucial step forward.

Another key discussion point was the role of disease severity in MLTC research. Participants noted that current methodologies often fail to distinguish between varying severities of conditions, which can distort assessments of disease burden. This point supports calls for more nuanced classification systems that incorporate disease severity, a gap also noted by Ho et al.,^42^ in their study of MLTC methodologies.

Transparency and reproducibility in MLTC research were identified as critical areas for improvement. Participants endorsed the development of specific reporting guidelines, similar to the EQUATOR guidelines,^43^ to standardise practice in this field to improve the reporting of data collection and analytical approaches. This could be developed through a Delphi consensus process, involving experts across various sectors. This reflects broader concerns in health research regarding the reproducibility crisis, particularly in complex, multifaceted fields such as MLTC.^44^

The importance of data sharing was another key theme. Participants advocated for a centralised data repository or MLTC data commons to facilitate collaboration and improve research efficiency. However, concerns surrounding data privacy, ethical approvals, and the complexity of linking different datasets were acknowledged as significant barriers, aligning with previous findings by Rosenbloom et al.^45^ The establishment of a secure, ethically governed national MLTC data repository was proposed as a potential solution to enable broader data access while ensuring compliance with regulatory frameworks. The integration of PPIE in MLTC research was widely supported, reinforcing the findings of existing literature stressing the benefits of patient involvement in improving research relevance and impact.^46^ Workshop participants highlighted that patients, carers, and healthcare professionals should play a central role in defining research priorities and shaping methodological approaches. This is consistent with findings from the James Lind Alliance Priority Setting,^47^ which stress the need for collaborative research agendas that reflect the lived experiences of individuals with MLTC.

A key gap identified was the lack of dedicated training and mentorship opportunities in MLTC research. Compared with single-disease research fields MLTC lacks structured professional networks and capacity-building initiatives. Participants called for the development of mentorship programmes to support early-career researchers and foster interdisciplinary expertise. The need for training in emerging methodologies, such as artificial intelligence (AI) and health technologies, was also emphasised, echoing calls for expanded training in interdisciplinary research techniques.^48,49^

Collaboration between academic and non-academic sectors was highlighted as essential for advancing MLTC research. Participants stressed the importance of involving policymakers, healthcare providers, and industry partners in research planning and execution. This approach aligns with Greenhalgh et al.,^50^ who advocate for stakeholder engagement to ensure research is practical and applicable in real-world healthcare settings. Additionally, the establishment of an MLTC research network or resource hub was proposed to facilitate knowledge exchange, funding updates, and methodological guidance. Similar initiatives in other health research fields have successfully fostered collaboration and standardisation of best practices.^51,52^

### Strengths and limitations

This workshop successfully brought together a range of diverse stakeholders, fostering interdisciplinary collaboration and ensuring that recommendations were practical and relevant to the wider MLTC research community. The structured discussions allowed prioritisation of key methodological improvements, while inclusion of patient and public involvement (PPI) strengthened the real-world applicability of findings. In particular, PPI were key in shaping the workshop objectives and structure, and they also influenced the context and direction of discussions, ensuring that the final synthesis of findings incorporated the lived experience of people living with MLTC and were relevant to their needs.^53–59^

However, the relatively small sample size and UK-centric focus may limit the generalisability of the findings. Although efforts were made to include diverse expertise, broader international participation would enhance the global relevance of future work. Although we had a diverse representation, further input from a broader range of sectors, such as public health experts, health economists, technology developers, legal and ethical professionals, and additional community organisations, could have strengthened the depth of the discussions. We acknowledge that this lack of wider sectoral representation may partly result from our ‘open call’ approach to participant recruitment (primarily conducted through established MLTC networks and organisations), which could have overly narrowed our sampling frame. A broader-based and systems-wide recruitment strategy may have increased the range and diversity of participants involved in the research.

Additionally, while key gaps were identified, further research, such as Delphi consensus studies or systematic reviews, is needed to refine and implement the proposed recommendations. Data sharing challenges and research network sustainability also require long-term institutional support and policy alignment.

### Conclusion

This work contributes to the evolving discourse on the methodological advancement of research into MLTC through the integration of diverse perspectives on this topic. The workshop generated a series of clear recommendations and action points aimed at enhancing MLTC research methodologies, fostering collaboration and ensuring methodological rigour. Further, our work provides insights for implementation into research and care practice, including the need for:

• application of consensus-driven standardised core condition lists and consistent outcome measurement approaches relevant to both research and care settings, to improve comparability and clarity across studies and evaluation of the efficacy of new care interventions;
• development of transparent and standardised reporting guidelines to enhance reproducibility, especially in the reporting of data use and selection criteria;
• greater data-sharing infrastructure to facilitate collaboration including a resource hub or repository to facilitate knowledge exchange and embed methodological advancements more widely;
• recognising the impact of disease severity and understanding the cause and effect of specific disease combinations, which can improve risk assessment profiling and the implementation of more personalised and effective care models;
• establishing an MLTC research network to strengthen patient and public involvement and wider stakeholder engagement, along with provision of training and mentorship opportunities, to enhance the relevance and impact of MLTC research and its implementation into care practice.

These findings provide a foundation for the continued advancement of MLTC research and the development of standardised approaches to measuring and analysing multiple long-term conditions. Additionally, the insights gained from this workshop highlight the importance of coordinated efforts across academia, healthcare, industry, and public engagement to enhance the quality and impact of research in this field and ensure that care planning and decision-making reflect the complex realities of MLTC. By addressing these methodological gaps and facilitating collaboration across disciplines through greater alignment of MLTC research with care practice, the MLTC research community can generate more rigorous, inclusive, and impactful evidence, ultimately improving care delivery and patient outcomes.

## Supplemental Material

Supplemental Material - Addressing methodological challenges in multiple long-term conditions research: A stakeholder workshop using a nominal group technique method Supplemental Material for Addressing methodological challenges in multiple long-term conditions research: A stakeholder workshop using a nominal group technique method by Hajira Dambha-Miller, Glenn Simpson, Lucy Smith, James Finney, Salwa S. Zghebi, Sarah E. Hughes, Victoria L Keevil, Ge Yu, Clare MacRae, Kamlesh Khunti, Colin McCowan, on behalf of the NIHR MLTC Cross-NIHR Collaboration Methodologies workstream in Journal of Multimorbidity and Comorbidity
