# Supplementary material for: Addressing methodological challenges in multiple long-term conditions research: A stakeholder workshop using a nominal group technique method
Source: J Multimorb Comorb. 2025 Sep 24;15:26335565251372222. doi: 10.1177/26335565251372222 (PMC12461038; doi:10.1177/26335565251372222)
Supplement: Supplemental Material - Addressing methodological challenges in multiple long-term conditions research: A stakeholder workshop using a nominal group technique method [file sj-pdf-1-cob-10.1177_26335565251372222.pdf]

Appendices

Appendix One - Participants by sector

Sector and profession of participants	Number of participants in each sector
Clinician/care professional/public health practitioner	7
Statistics/data science academic	5
Programme manager	2
Epidemiologist	2
PPI representative	2
Industry representative	1
Policy professional	1
Health economist	1
Methodologist	1
Theoretical Physicist	1
Total	*23*

Appendix Two - Participants - academic institutions and affiliations

- Participant 1, University of St Andrews
- Participant 2, University of Southampton
- Participant 3, King’s College London
- Participant 4, University of Leeds
- Participant 5, King’s College London
- Participant 6, University of Newcastle
- Participant 7, University of Oxford
- Participant 8, University of Liverpool
- Participant 9, University of Swansea
- Participant 10, University of Manchester
- Participant 11, University of Birmingham
- Participant 12, University of Cambridge
- Participant 13, University of Leicester
- Participant 14, University of Newcastle
- Participant 15, No institutional affiliation
- Participant 16, No institutional affiliation
- Participant 17, University of Edinburgh
- Participant 18, University of Swansea
- Participant 19, University of Newcastle
- Participant 20, Office of National Statistics
- Participant 21, University of Warwick
- Participant 22, Innovate UK Business Connect
- Participant 23, University of Leicester

Appendix Three – Open call circulation list for recruitment of study participants

- ***NIHR networks***
    - NIHR ARC East Midlands.
    - NIHR Newcastle BRC.
    - NIHR Central Coordinating Facility.
    - NIHR Academy.
- ***Academic Institutions***
    - The NIHR MLTC CNC programme has approximately 60 members from around 20 different institutions. The call was shared across members' respective personal networks.
- ***Social media platforms***
    - NIHR MLTC CNC – Linked-In page
    - X (Twitter) – NIHR ARC East Midlands and NIHR Newcastle BRC accounts.
    - Bluesky – NIHR ARC East Midlands and NIHR Newcastle BRC accounts.
- ***Partner organisations***
    - Innovate UK Business Connect.
    - Royal College of General Practitioners.
    - Royal College of Physicians.

Appendix Four - Structured Discussion Guide

Workshop Topic Guide

Overall purpose: To explore methodological challenges in Multiple Long-Term Conditions (MLTC) research and collaboratively develop practical, actionable solutions through structured, cross-disciplinary dialogue.

Session 1: Contextualisation and Thematic Identification

Purpose of the session

To develop a shared understanding of methodological challenges in MLTC research and identify key themes for deeper exploration.

1. Opening and welcome

Facilitator guidance:

Introduce the aims and structure of the workshop. Emphasise collaborative working, diversity of experience, and inclusive participation.

2. Setting the scene: expert presentations

Facilitator guidance:

Invite experts to provide an overview of current MLTC research challenges. Present theoretical and practical issues to establish a shared context.

3. Clarifying the scope of MLTC methodology

Facilitator guidance:

Summarise issues raised in the presentations, particularly around variation in definitions, inclusion criteria, and methodological approaches.

4. Highlighting gaps in current methods

Facilitator guidance:

Encourage reflection on current research practices and where they fail to adequately capture the complexity of MLTC.

5. Brainstorming methodological challenges

Facilitator guidance:

Facilitate an open brainstorming activity. Ensure wide input from all sectors and backgrounds, including patients and carers.

6. Capturing and organising ideas

Facilitator guidance:

Document all contributions visibly using flipcharts or digital tools. Begin to loosely group similar ideas without filtering content.

7. Thematic clustering of contributions

Facilitator guidance:

Work with participants to group ideas into initial themes. Highlight any emerging patterns or areas of agreement.

8. Refining and naming themes

Facilitator guidance:

Facilitate discussion to refine and label themes. Ensure language is accessible and that clusters reflect the full range of contributions.

9. Agreeing priority themes for session 2

Facilitator guidance:

Support the group in selecting priority themes to take forward. Ensure that choices reflect both relevance and potential for solution development.

10. Summary and transition

Facilitator guidance:

Confirm the three agreed themes and explain the structure for Session 2. Let the participants know they will be assigned to groups based on experience and expertise.

Session 2: Thematic Exploration and Solution Development

Purpose of the session

To explore each priority theme in depth and collaboratively generate practical, evidence-informed solutions to specific methodological challenges.

1. Introduction to thematic groups

Facilitator guidance:

Explain the rationale for dividing participants into smaller groups. Each group will focus on one of the three themes identified in Session 1. Groups have been pre-assigned to ensure a balanced mix of perspectives and expertise.

2. Structured group work begins

Facilitator guidance:

Facilitators use a structured discussion guide to support exploration of each theme. Encourage equal participation and maintain focus on specific methodological issues.

3. Exploring the theme in depth

Facilitator guidance:

Use structured techniques such as issue mapping, root cause analysis, or problem trees to break down the challenge. Support the participants in identifying the key contributing factors and barriers.

4. Generating and refining ideas

Facilitator guidance:

Encourage creative but realistic solution-building. Use iterative discussion to develop, refine,

and test ideas. Ensure all suggestions are grounded in practical feasibility and research relevance.

5. Synthesising group outputs

Facilitator guidance:

Work with the group to synthesise contributions into a small number of clearly defined, actionable recommendations. Ensure alignment with the theme and workshop goals.

6. Preparing to share back

Facilitator guidance:

Summarise key discussion points and final outputs. Prepare a short, clear summary of recommendations to present.

Appendix Five - ACCORD Checklist

Item No.	Section	Checklist Item (*help text*)	Page No.
T1	**Title**	Identify the article as reporting a consensus exercise and state the consensus methods used in the title.<br>*For example, Delphi or nominal group technique.*	Title page
I1	**Introduction**	Explain why a consensus exercise was chosen over other approaches.	5
I2		State the aim of the consensus exercise, including its intended audience and geographical scope (national, regional, global).	4
I3		If the consensus exercise is an update of an existing document, state why an update is needed, and provide the citation for the original document.	Not Applicable
M1	**Methods Registration**	If the study or study protocol was prospectively registered, state the registration platform and provide a link. If the exercise was not registered, this should be stated.<br>*Recommended to include the date of registration.*	Not Registered
M2	**Selection of SC and/or panellists**	Describe the role(s) and areas of expertise or experience of those directing the consensus exercise.<br>*For example, whether the project was led by a chair, co-chairs or a steering committee, and, if so, how they were chosen. List their names if appropriate, and whether there were any subgroups for individual steps in the process.*	5
M3		Explain the criteria for panellist inclusion and the rationale for panellist numbers. State who was responsible for panellist selection.	4
M4		Describe the recruitment process (how panellists were invited to participate).<br>*Include communication/advertisement method(s) and locations, numbers of invitations sent, and whether there was centralised oversight of invitations or if panellists were asked/allowed to suggest other members of the panel.*	4
M5		Describe the role of any members of the public, patients or carers in the different steps of the study.	4,7
M6	**Preparatory research**	Describe how information was obtained prior to generating items or other materials used during the consensus exercise.<br>*This might include a literature review, interviews, surveys, or another process.*	5
M7		Describe any systematic literature search in detail, including the search strategy and dates of search or the citation if published already.<br>*Provide the details suggested by the reporting guideline PRISMA and the related PRISMA-Search extension.*	N/A
M8		Describe how any existing scientific evidence was summarised and if this evidence was provided to the	5

		panellists.	
M9	**Assessing consensus**	Describe the methods used and steps taken to gather panellist input and reach consensus (for example, Delphi, RAND-UCLA, nominal group technique).<br>*If modifications were made to the method in its original form, provide a detailed explanation of how the method was adjusted and why this was necessary for the purpose of your consensus-based study.*	5-6
M10		Describe how each question or statement was presented and the response options. State whether panellists were able to or required to explain their responses, and whether they could propose new items.<br>*Where possible, present the questionnaire or list of statements as supplementary material.*	5-7
M11		State the objective of each consensus step.<br>*A step could be a consensus meeting, a discussion or interview session, or a Delphi round.*	7-10
M12		State the definition of consensus (for example, number, percentage, or categorical rating, such as 'agree' or 'strongly agree') and explain the rationale for that definition.	5
M13		State whether items that met the prespecified definition of consensus were included in any subsequent voting rounds.	N/A
M14		For each step, describe how responses were collected, and whether responses were collected in a group setting or individually.	4-7
M15		Describe how responses were processed and/or synthesised.<br>*Include qualitative analyses of free-text responses (for example, thematic, content or cluster analysis) and/or quantitative analytical methods, if used.*	5-7
M16		Describe any piloting of the study materials and/or survey instruments.<br>*Include how many individuals piloted the study materials, the rationale for the selection of those individuals, any changes made as a result and whether their responses were used in the calculation of the final consensus. If no pilot was conducted, this should be stated.*	N/A.
M17		If applicable, describe how feedback was provided to panellists at the end of each consensus step or meeting.<br>*State whether feedback was quantitative (for example, approval rates per topic/item) and/or qualitative (for example, comments, or lists of approved items), and whether it was anonymised.*	5,7
M18		State whether anonymity was planned in the study design. Explain where and to whom it was applied and what methods were used to guarantee anonymity.	N/A
M19		State if the steering committee was involved in the decisions made by the consensus panel.<br>*For example, whether the steering committee or those managing consensus also had voting rights.*	N/A
M20	**Participation**	Describe any incentives used to encourage responses or participation in the consensus process.<br>*For example, were invitations to participate reiterated, or were participants reimbursed for their time.*	N/A
M21		Describe any adaptations to make the surveys/meetings more accessible.	6

		For example, the languages in which the surveys/meetings were conducted and whether translations or plain language summaries were available.	
R1	**Results**	State when the consensus exercise was conducted. List the date of initiation and the time taken to complete each consensus step, analysis, and any extensions or delays in the analysis.	4
R2		Explain any deviations from the study protocol, and why these were necessary. *For example, addition of panel members during the exercise, number of consensus steps, stopping criteria; report the step(s) in which this occurred.*	N/A
R3		For each step, report quantitative (number of panellists, response rate) and qualitative (relevant socio-demographics) data to describe the participating panellists.	7
R4		Report the final outcome of the consensus process as qualitative (for example, aggregated themes from comments) and/or quantitative (for example, summary statistics, score means, medians and/or ranges) data.	7-10
R5		List any items or topics that were modified or removed during the consensus process. Include why and when in the process they were modified or removed.	N/A
D1	**Discussion**	Discuss the methodological strengths and limitations of the consensus exercise. *Include factors that may have impacted the decisions (for example, response rates, representativeness of the panel, potential for feedback during consensus to bias responses, potential impact of any non-anonymised interactions).*	13
D2		Discuss whether the recommendations are consistent with any pre-existing literature and, if not, propose reasons why this process may have arrived at alternative conclusions.	10-12
O1	**Other information**	List any endorsing organisations involved and their role.	3,14
O2		State any potential conflicts of interests, including among those directing the consensus study and panellists. Describe how conflicts of interest were managed.	14
O3		State any funding received and the role of the funder. *Specify, for example, any funder involvement in the study concept/design, participation in the steering committee, conducting the consensus process, funding of any medical writing support. This could be disclosed in the methods or in the relevant transparency section of the manuscript. Where a funder did not play a role in the process or influence the decisions reached, this should be specified.*	1,14
